# Systemic Immune‐Inflammation Index and Gait Speed in Older Adults: Evidence From NHANES 1999–2002

**DOI:** 10.1002/kjm2.70270

**Published:** 2026-07-29

**Authors:** Ying‐Lun Chen, Zhen‐Xuan Ma

**Affiliations:** ^1^ Department of Rehabilitation Medicine Shanghai Geriatric Medical Center Shanghai China; ^2^ Department of Epidemiology and Biostatistics Indiana University Bloomington Indiana USA

**Abbreviations**
NHANES: The National Health and Nutrition Examination Survey
SII: systemic immune‐inflammation index

Dear Editor,

Reduced gait speed, often described as the “sixth vital sign,” is a well‐established indicator of functional decline and adverse health outcomes in older adults [1]. Chronic low‐grade inflammation has been implicated in age‐related deterioration of physical performance. However, the relationship between the systemic immune‐inflammation index (SII)—a composite marker derived from platelet, neutrophil, and lymphocyte counts, and gait speed—remains unknown. Therefore, we aimed to investigate the association between SII and gait speed in older adults and explore the potential mediating roles of muscle strength and lower‐limb body composition.

We analyzed data from 2007 community‐dwelling adults aged 60 years or older who participated in the National Health and Nutrition Examination Survey (NHANES) 1999–2002. SII was calculated as platelet count × neutrophil count/lymphocyte count. Gait speed was assessed using a standardized 20‐ft walk test. Knee extensor strength, calf circumference, and relative lower appendicular lean mass were evaluated as muscle‐related indicators. Survey‐weighted multivariable regression models were adjusted for demographic characteristics, socioeconomic status, smoking status, body mass index, hypertension, and diabetes. Potential pathways linking SII to gait speed were identified using mediation analyses.

The key findings are summarized in Table 1. The mean age of the participants was 71 years, and 49.9% were women. Higher SII levels were independently associated with slower gait speed after adjusting for covariates (*β* = −0.028, SE = 0.010, *p* = 0.044). Participants in the highest SII quartile demonstrated a significantly slower gait speed than those in the lowest quartile (*p* < 0.001). In sex‐stratified analyses, the association between SII and gait speed was more evident in men (*β* = −0.050, SE = 0.015, *p* = 0.004). Mediation analyses suggested elevated SII was associated with lower knee extensor strength and reduced relative lower appendicular lean mass. Knee extensor strength and calf circumference partially explained the association between SII and gait speed, accounting for 32.1% (mediation effect = −1.62 × 10^−5^, *p* < 0.001) and 13.6% (mediation effect = −5.53 × 10^−6^, *p* = 0.014) of the total effect, respectively.

**TABLE 1:** Associations of SII levels with gait indicators and the mediation roles of muscle‐related indicators.

	Gait speed		Muscle strength		Calf circumference		Relative lower appendicular lean mass	
	*β* (std. error)	*p*	*β* (std. error)	*p*	*β* (std. error)	*p*	*β* (std. error)	*p*
log (SII)	−0.028 (0.010)	0.044*	0.001 (0.000)	0.001**	0.010 (0.003)	0.028*	−0.100 (0.388)	0.810
Total								
Q2	0.007 (0.019)	0.723	−5.36 (6.51)	0.423	0.147 (0.184)	0.438	−0.003 (0.001)	0.094^+^
Q3	0.001 (0.019)	0.969	−12.3 (7.32)	0.113	0.415 (0.215)	0.071^+^	−0.003 (0.001)	0.045*
Q4	−0.034 (0.014)	0.028*	−21.1 (5.41)	0.001*	−0.134 (0.146)	0.373	−0.005 (0.001)	< 0.001**
P trend	< 0.001**		< 0.001**		0.012*		0.021*	
Man								
Q2	−0.001 (0.023)	0.953	−10.3 (10.0)	0.319	0.203 (0.245)	0.419	−0.002 (0.002)	0.319
Q3	−0.001 (0.024)	0.970	−2.37 (9.94)	0.814	0.465 (0.223)	0.053^+^	−0.002 (0.002)	0.433
Q4	−0.050 (0.015)	0.004*	−25.6 (7.68)	0.004*	−0.033 (0.210)	0.876	−0.006 (0.002)	0.015*
P trend	< 0.001**		< 0.001**		0.009*		0.158	
Women								
Q2	0.012 (0.033)	0.731	−0.333 (9.25)	0.971	0.114 (0.330)	0.734	−0.003 (0.003)	0.295
Q3	0.0004 (0.028)	0.986	−14.2 (8.99)	0.132	0.377 (0.346)	0.290	−0.004 (0.002)	0.052^+^
Q4	−0.023 (0.023)	0.326	−17.1 (6.52)	0.018*	−0.220 (0.280)	0.444	−0.006 (0.002)	0.006*
P trend	0.087^+^		0.044*		0.095^+^		0.004*	

*Note:* SII was divided into 4 groups based on quartile (Q1, Q2, Q3, Q4). Q1 serves as the reference group. Q1 (< 368), Q2 (368–598), Q3 (598–715), Q4 (> 715). p*** means *p* value < 0.001, p* means *p* value < 0.05, *p* ^+^ means *p* value < 0.10.

Abbreviation: SII, systemic immune‐inflammation index.

These findings suggest that systemic inflammatory status may be associated with lower gait speed in older adults, potentially through molecular pathways involving muscle strength and defining lower‐limb muscle characteristics rather than muscle mass alone. Chronic inflammation may contribute to reduced muscle protein synthesis and increased muscle catabolism, thereby adversely affecting physical performance [2]. Inflammatory processes may influence neuromuscular function and connective tissue properties. Inflammatory processes affect muscle quality and neuromuscular performance beyond reduction in muscle quantity [3]. Sex‐related differences were observed in the association between systemic inflammation and lower‐limb muscle strength, with stronger associations in men than women. Sex‐related differences may be explained by differences in muscle fiber composition, hormonal regulation, and age‐related muscle loss [4]. Estrogen exerts a protective effect on muscle metabolism and inflammatory responses [5]. Sex‐specific mechanisms underlying inflammation‐related deterioration of physical performance with increasing age remain unclear and warrant further investigation. Although causality cannot be inferred from this cross‐sectional analysis, our results support the potential utility of SII as an accessible biomarker for identifying older individuals at risk of mobility impairment. Longitudinal studies are warranted to clarify the temporal relationships and underlying biological mechanisms.

## Funding

This work was supported by Clinical Research Project of Shanghai Municipal Health Commission [grant no. 20244Y0064] and Shanghai Institute of Rehabilitation with Integrated Western and Chinese Traditional Medicine [grant no. 2025XKPT25‐RC1].

## Ethics Statement

The NHANES study was approved by the National Center for Health Statistics Research Ethics Review Board, with publicly accessible datasets (https://www.cdc.gov/nchs/nhanes/index.htm). The current study is a population‐based, cross‐sectional study and is not considered a clinical trial. All procedures performed in this study were performed under the ethical standards of the national research committee and in agreement with the Helsinki Declaration. Informed consent to participate was obtained from all of the participants. Written informed consent has been obtained from the patient(s) to publish this paper.

## Conflicts of Interest

The authors declare no conflicts of interest.

## Data Availability

The NHANES data used in this study are publicly available and can be accessed at the following website: https://wwwn.cdc.gov/nchs/nhanes.
